# Skull Base Repair following Resection of Vestibular Schwannoma: A Systematic Review (Part 2: The Translabyrinthine Approach)

**DOI:** 10.1055/a-2222-0016

**Published:** 2024-01-22

**Authors:** Joachim Starup-Hansen, Simon C. Williams, Alexandra Valetopoulou, Danyal Z. Khan, Hugo Layard Horsfall, Jigishaa Moudgil-Joshi, Oliver Burton, Hala Kanona, Shakeel R. Saeed, William Muirhead, Hani J. Marcus, Patrick Grover

**Affiliations:** 1 Victor Horsley Department of Neurosurgery, The National Hospital for Neurology and Neurosurgery, University College London NHS Trust, London, United Kingdom; 2 Wellcome/EPSRC Centre for Interventional and Surgical Sciences, University College London, London, United Kingdom; 3 Department of Neurosurgery, The Royal London Hospital, London United Kingdom; 4 Department of Neurosurgery, Charing Cross Hospital, London, United Kingdom; 5 The Royal National Throat, Nose and Ear Hospital, London, United Kingdom; 6 University College London Ear Institute, London, United Kingdom

**Keywords:** vestibular schwannoma, CSF leak, skull base repair, neurosurgery, ear nose and throat, translabyrinthine approach

## Abstract

**Objectives** Despite advances in skull base reconstruction techniques, cerebrospinal fluid (CSF) leaks remain a relatively common complication after translabyrinthine (TL) vestibular schwannoma (VS) surgery. We conducted a systematic review to synthesize the repair techniques and materials used in TL VS surgery to prevent CSF leaks.

**Design** A systematic review of studies published since 2000 reporting techniques to prevent CSF leaks during adult TL VS surgery was conducted. A narrative synthesis of primary repair protocols was produced, and a taxonomy was established. Additionally, the advantages, disadvantages, and associated CSF leak rates of different repair protocols were extracted.

**Results** All 43 studies were case series, and 39 were retrospective. Repair strategies included heterogeneous combinations of autografts, xenografts, and synthetic materials. A taxonomy was produced, classifying repairs into seven distinct stages, including approaches to the dura, middle ear cleft, air cells, TL bony defect, extra-cranial soft tissue, postoperative dressings, and CSF diversion. The median postoperative incidence of CSF leaks was 6% (interquartile range: 0–10%).

**Conclusions** This systematic review reveals substantial inter-institutional heterogeneity in intraoperative strategies to prevent CSF leaks following TL VS surgery. However, comparing these techniques is challenging due to the multiple predictive factors for CSF leaks and their inconsistent reporting. We propose a taxonomy of seven stages to classify operative techniques and materials aimed at preventing CSF leaks. We recommend that future evaluations should adopt a prospective approach encompassing data collection strategies that considers all operative stages described by our taxonomy.

## Introduction

The translabyrinthine (TL) approach is a common surgical approach to the cerebellopontine angle, primarily used for the resection of vestibular schwannomas (VSs), in patients who have lost serviceable hearing. 1 The morbidity and mortality from VS surgery have improved significantly over the past 50 years, in part through advances in operative techniques. 2 3 However, surgical resection via the TL approach by definition involves disruption of the lateral skull base, introducing the risk of complications. The most common complication following TL VS surgery is the leakage of cerebrospinal fluid (CSF), occurring in 10% of cases. 4 The implications of CSF leaks are potentially serious, including life-threatening meningitis, wound infections, prolonged hospitalization, repeated interventions, and an augmented burden on healthcare expenditures. 5 6 7

Several factors influence the incidence of postoperative CSF leaks, including patient factors, choice of approach, and the operative method of skull base reconstruction. 8 9 10 11 While certain factors are non-modifiable, the strategy used to repair the skull base remains within the surgeon's sphere of influence. Indeed, an array of surgical techniques using a variety of biomaterials, such as autografts, xenografts, and synthetic substitutes, have been developed with the intention of restoring the multiple anatomical defects created during surgery. Additionally, pressure-reducing strategies via CSF diversion (i.e., lumbar drains) may also be used. 12 As a result of the many strategies available, the optimal combination of techniques and materials remains unclear.

To determine the optimal protocol for preventing CSF leaks, an overview is required to organize the many available options reported in the literature. The present systematic review provides a taxonomic classification of skull base repair strategies following VS resection performed via the TL approach. Herewith, we aim to elucidate the advantages, disadvantages, and outcomes associated with each repair technique, assisting surgeons in making informed decisions and guiding future prospective service evaluations.

## Methods

A PRISMA adherent systematic review of the literature was performed. This publication is part 2 of a two-part series, considering skull base repair techniques for VS surgery via the retrosigmoid and TL approaches, respectively. A study protocol was generated prior to data collection (PROSPERO ID: CRD42023388780).

### Search Strategy

The search strategy comprised synonymous terms for “CSF leak” and “vestibular schwannoma,” a full search strategy can be found in Supplementary Table S1 (available in the online version only). To be included in the analysis, studies had to (1) be published in English from 2000 to 2023; (2) report a technique for skull base repair following the resection of VSs via the TL approach; and (3) include the incidence of postoperative CSF leakage. Exclusion criteria were case series with fewer than three VS patients, conference abstracts, editorials, reviews, animal studies, and cadaveric studies. Studies that reported multiple surgical approaches (e.g., retrosigmoid approach, middle fossa approach) were included only if they reported CSF-related outcomes for the TL approach separately; articles that provided combined leak rates of different surgical approaches were excluded. Studies reporting non-VS indications for TL surgery were included if VSs made up at least three cases (consistent with our case series limit). If author groups published multiple studies reporting on an identical cohort of patients, the study with the most recent results was included, and prior studies were excluded to avoid duplicate results. PubMed and EMBASE databases were searched on March 15, 2023. Citation references of included studies were reviewed for additional candidate articles.

OVID and Rayyan (version 9.4.1) were used for de-duplication. Abstract screening was conducted by two independent reviewers (J.S.-H., S.C.W.). Any conflicts between reviewers were resolved through arbitration by a third author (H.J.M.).

### Data Extraction

Extracted data points of included studies consisted of study details (design, follow-up length), patient demographics (e.g., sample size, age, and sex), tumors characteristics (size), CSF preventative strategies (techniques, materials), strategy rationales, CSF leak identification strategies, CSF leak rates, and the treatment strategies following confirmation of CSF leaks. If studies reported multiple techniques with individual cohort descriptions, this was reflected in the data extraction.

### Quality Assessment

Risk of bias was analyzed using a bespoke tool adapted from a prior systematic review of endonasal reconstructive strategies conducted by our group ( Supplementary Table S2 , available in the online version only). 13 The tool is based on COSMOS-E guidance 14 and interrogates key study properties including the clarity of reporting of CSF leak risk factors, treatment groups, repair strategies, and outcome definitions. Studies were rated out of 5 and stratified according to lowest risk (score 0–1) and highest risk (score 4–5).

### Data Analysis

Data were analyzed in Excel (Microsoft, United States, version 16.66.1) and combined into a narrative synthesis, delineating the techniques and materials used to prevent CSF leaks following VS TL surgery and their associated frequency. Additionally, a taxonomic classification of repair strategies, with subgroupings based on the anatomic level of repair, was produced. However, given the heterogeneity of repair protocols across studies, no attempt was made to comment on the superiority of the various strategies—except in circumstances where individual studies identified statistically significant drawbacks to a technique. The incidence of CSF leaks was analyzed using descriptive statistics (median, interquartile range [IQR]) to account for the heterogenous inclusion criteria and possible overlap of patient cohorts by some groups, limiting the validity of a pooled synthesis.

## Results

### Overview

**Fig. 1:**
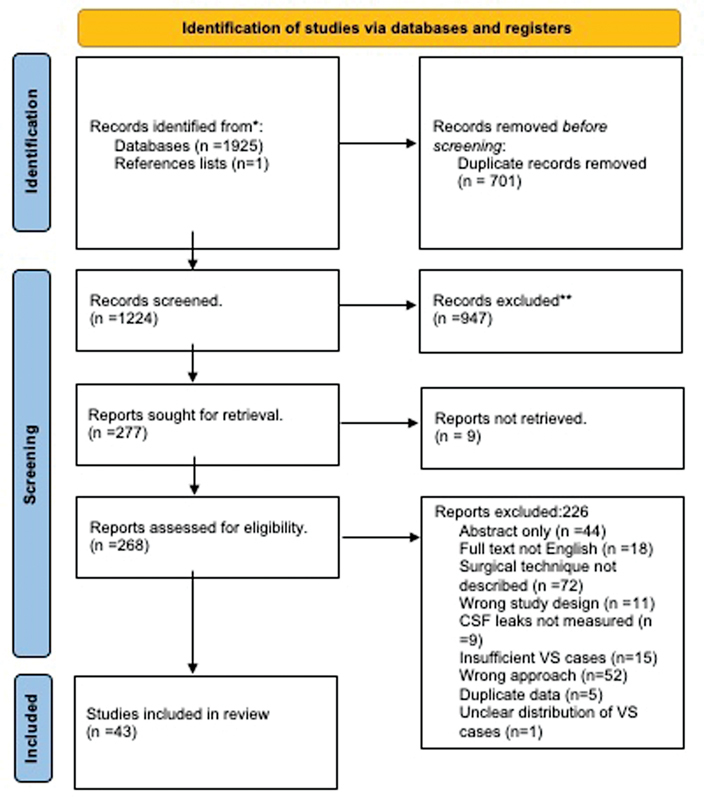
PRISMA flow chart describing how articles were selected for inclusion in the final analysis.

The search identified 1,925 articles, and 43 studies were included in the full-text analysis (see Fig. 1 ). Table 1 provides a synthesis of study demographics. Most studies were retrospective (39/43), and all were case series. Four studies had at least one arm which was prospective. 15 16 17 18 Twelve out of 43 studies compared different techniques or materials. 15 19 20 21 22 23 24 25 26 27 28 29 Four studies described non-VS indications for TL surgery, which included facial nerve schwannomas, epidermoid tumors, meningiomas, paragangliomas, and other nonspecified tumors (see Table 1 ). 15 25 30 31 The median risk of bias was 2/5 (IQR: 1–3), suggesting moderate risk of bias ( Supplementary Table S3 [available in the online version only]). Most groups were from North America (29/43, 67%), followed by Europe (11/43, 26%), Asia (1/43, 2%), Africa (1/43, 2%), and South America (1/43, 2%).

**Table 1:** Study demographics, ordered by study design and year of publication

Authors	Continent	Year	Study type	Subtypes of repair technique	Total patients	Age in years (mean)	Sex (M:F)	Pathologies	Mean tumor size (mm)	BMI	Follow-up
Kalamarides et al 18	Europe	2004	Prospective case series		139	NS	NS	VS	NS	NS	1 y +
Sen et al 16	Europe	2006	Prospective case series		24	51	12 M:12 F	VS	NS	NS	NS
Moderie et al 17	North America	2017	Prospective case series		94	35.5	40 M:54 F	VS	24.1	NS	2.1 y
Hillman and Shelton 15	North America	2011	Prospective case series with matched cohort analysis	*TL cavity: fat + resorbable plate*	71	51	34 M:37 F	*n* = 70 VS, *n* = 1 meningioma	20	NS	NS
				TL cavity: fat closure	149	49.8 a	68 M:81 F	*n* = 147 VS, *n* = 2 meningioma	22	NS	NS
Brennan et al 57	North America	2001	Retrospective case series		431	NS	NS	VS	21.3	NS	NS
Leonetti et al 31	North America	2001	Retrospective case series		209	56.4	1 M: 1.18 F	VS 77%, meningioma 10%, paraganglioma 7%, other 6%	<1 cm 23%, 1.1–2.0 cm 17%, 2.1–3 cm 40%, >3 cm 20%	NS	NS
Arriaga and Chen 21	North America	2002	Retrospective case series	*AFG*	54	NS	26 M:28 F	VS	22	NS	1 y+
				*HAC 1 (hardened cement contacts dura*	7	NS	24 M:30 F	VS			NS
				*HAC 2 (fat contacts dura, then filled with HAC)*	47	NS					
Becker et al 10	North America	2003	Retrospective case series		100	51	50 M:50 F	VS	22	NS	NS
Sanna et al 32	Europe	2003	Retrospective case series		596	NS	NS	VS	NS	NS	NS
Cueva and Mastrodimos 64	North America	2005	Retrospective case series		126	NS	NS	VS	20	NS	NS
Hussain and Ahsan 46	North America	2005	Retrospective case series		20	54.4	7 M:3 F	VS	NS		6 mo
Arriaga et al 38	North America	2007	Retrospective case series		90	NS	NS		22		
Fayad et al 41	North America	2007	Retrospective case series		389	47.6	187 M: 202 F	VS	24	NS	NS
Jacob et al 19	North America	2007	Retrospective case series	ET packing	148	NS	NS	NS	NS	NS	NS
	North America	2007	Retrospective case series	*No ET packing*	51	NS	NS	NS	NS	NS	NS
Roche et al 37	Europe	2008	Retrospective case series		110	50.1	44 M:66 F	VS	Stage IV (KOOS)	NS	NS
Yuen et al 29	North America	2009	Retrospective case series	*TL cavity: vascularized mastoid bone flap*	17	56.5	7 M:10 F	VS	30	NS	12–15 mo
				*TL cavity: fat packing*	10	51.3	6 M:4 F	VS	28	NS	1 y+
Charpiot et al 59	Europe	2009	Retrospective case series		123	46.4	62 M:61 F	VS	Stage IV KOOS	NS	1 y+
Wiet et al 39	North America	2000	Retrospective case series		44	57.8	21 M:23 F	VS	19	NS	NS
Khrais et al 33	Europe	2004	Retrospective case series		710	50.5	332 M:378 F	VS	20		NS
Fishman et al 40	North America	2004	Retrospective case series		101	NS	NS	VS	NS	NS	NS
Goddard et al 35	North America	2010	Retrospective case series		61	46.6	27 M:34 F	VS	23	NS	31.3 mo
Bambakidis et al 50	North America	2010	Retrospective case series		15	53.5	7 M:8 F	VS	12 a	NS	NS
Angeli et al 54	Europe	2011	Retrospective case series		110	42.5	51 M:59 F	VS	41	NS	1 y+
Netto et al 28	South America	2012	Retrospective case series	*Dural repair: fat plugging*	18	51.47	13 M: 21 F	VS	23	NS	1 y+
				*Dural repair: synthetic dura + fat plugging*	16					NS	NS
Ben Ammar et al 34	Africa	2012	Retrospective case series		1,865	50.39	886 M:979 F	VS	18	NS	5.7 y
Liu et al 22	North America	2012	Retrospective case series	*Dural repair: fascial sling*	8	55.6	5 M:3 F	VS	NS	NS	13.8 mo
				*Dural repair: synthetic sling*	5	49.6	1 M:4 F	VS	NS	NS	35.8 mo
Zhang et al 58	Asia	2012	Retrospective case series		115	46.42	50 M:65 F	VS	40.7	NS	1 y+
Manjila et al 47	North America	2013	Retrospective case series		42	50.5	21 M:21 F	VS	15.1	NS	NS
Boghani et al 45	North America	2013	Retrospective case series		13	51.9 b	1 M:1.33 F b	VS	NS	NS	NS
Hunter et al 48	North America	2015	Retrospective case series		53	54	29 M:34 F	CPA tumors	18.8	30.8	5.4 mo
Crowson et al 12	North America	2016	Retrospective case series	*Lumbar drain*	110	52.3 b	1 M:1.33 F b	VS	19.1 b	NS	NS
	North America	2016	Retrospective case series	*No lumbar drain*	11	52.3 b	1 M:1.33 F b	VS	19.1 b	NS	NS
Volsky et al 36	North America	2017	Retrospective case series		369	NS	NS	VS	NS	NS	Pittsburgh 5.2 y, Louisiana 577 d
Russel et al 55	Europe	2017	Retrospective case series		275	55.4 a	122 M:153 F	VS	NS	25.3 a	NS
Ölander et al 56	Europe	2018	Retrospective case series		700	53	330 M:370 F	VS	27	NS	NS
Luryi et al 30	North America	2020	Retrospective case series		52	56.2	27 M:25 F	VS 51, meningioma 1	22	NS	30.5 mo
Plainfossé et al 23	Europe	2022	Retrospective case series	Without ET packing	8	53.6	4.55 M:1 F b	VS	NS	25.7	5.5 y
Plainfossé et al 23	Europe	2022	Retrospective case series	With ET packing	39	53.6					
Cooper et al 42	North America	2021	Retrospective case series		23	57	9 M:14 F	VS	NS	NS	21 mo
Totten et al 25	North America	2021	Retrospective case series	*Dural autograft*	56	49	NS	VS = 54, FN schwannoma = 2	22	27.7 ( a )	15 mo a
				*Dural xenograft*	21	47	NS	VS = 18, epidermoid = 2, meningioma = 1	25	27.7 ( a )	14 mo a
Selleck et al 27	North America	2021	Retrospective case series	TL cavity: fat only	38	56.4	13 M:25 F	VS	19.6	30.7	691 d
				*TL cavity: fat + plate*	94	51	40 M:54 F	VS	22	30.5	3,369 d
Martinez-Perez et al 24	Europe	2022	Retrospective case series	Tl cavity: mastoid fat + HAC	34	49.7	14 M:20 F	CPA tumors	NS	NS	NS
			Retrospective case series	TL cavity: mastoid fat	35	50.7	17 M:18 F		NS	NS	NS
Sioshansi et al 20	North America	2021	Retrospective case series	*TL cavity: Fat Graft*	30	53.67	14 M:16 F	VS	19.36	27.5	3 mo+
				*TL cavity: HAC*	33	54.8	13 M:17 F	VS	20.6	28.9	3 mo +
Freeman et al 49	North America	2023	Retrospective case series		205	52.5	77 M:128 F	VS	23	28.8	NS
Christopher et al 26	North America	2021	Retrospective case series with matched cohort analysis	*Mastoid approach*	102	51	45 M: 57 F	VS	24.1	NS	NS
			Retrospective case series with matched cohort analysis	*Facial recess approach*	102	49	48 M:54 F	VS	24.2	NS	NS

Abbreviations: AAA, aditus ad antrum; BW, bone wax; CSF, cerebrospinal fluid; ET, eustachian tube; HAC, hydroxyapatite cement; LD, lumbar drain; ME, middle ear; NS, not specified; s/c, subcutaneous; TL, translabyrinthine; WT, watertight.

a Median.

b Value averaged from larger dataset.

### Repair Techniques

**Fig. 2:**
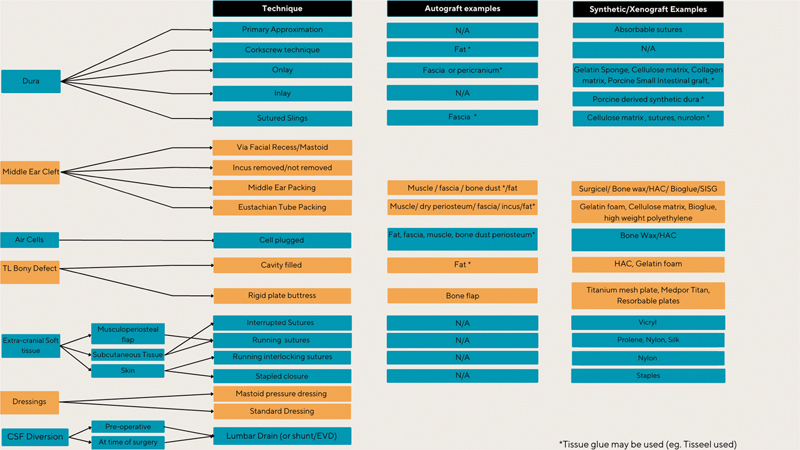
Taxonomy of translabyrinthine repair protocols. CSF, cerebrospinal fluid; EVD, external ventricular drain; HAC, hydroxyapatite cement; N/A, not applicable; TL, translabyrinthine.

There was significant heterogeneity in the skull base repair strategies, with no two author groups reporting identical techniques and materials in all stages of repair. Supplementary Table S3 (available in the online version only) offers an exhaustive review of the surgical techniques identified. All included studies focused on barrier restoring strategies to approach the skull base, with few studies reporting pressure-reducing strategies (i.e., lumbar drains). Regarding intra-study consistency, most studies performed the same technique in all cases. However, some reported variations between surgeons, guided by personal preference. 24 Overall, surgical repair techniques were rarely reported as being adapted to patient or anatomical factors. Exceptions to this included the degree of temporal bone pneumatization, which was used to guide middle ear (ME) treatment in two studies, 32 33 and the outcome of an intraoperative Valsalva, which prompted middle-ear and eustachian tube (ET) treatment, if CSF was found to collect in the mastoid antrum. 31 Repair strategies were taxonomized into seven anatomical stages of repair, produced in Fig. 2 .

### Dural Phase

The TL approach usually involves resection of the dura, making a primary watertight repair impossible. Therefore, various duraplasty techniques were described, including packing the defect with fat, applying an extradural “onlay,” or an intradural “inlay” graft with or without suturing.

Fat-packing, whereby strips of autologous fat typically harvested from the abdominal wall are inserted into the dural opening, also known as a “champagne cork technique,” was the most common technique for dural treatment 15 20 21 27 28 29 31 32 33 34 35 36 37 38 39 40 41 42 (references are not necessarily exhaustive). The seal mechanism proposed is that despite the autologous fat partially dissolving, it will form a pseudomembrane that maintains the dural-arachnoid seal during the formative stages of wound healing. Thirteen studies 15 20 21 27 28 31 32 33 35 36 37 38 39 42 performed fat packing in isolation with no reported adjuncts. Some institutions 40 41 42 attempted to mitigate the associated risks with overpacking, namely causing compression of critical structures, 22 43 44 by narrowing the dural opening through sutured approximation. Similarly, Netto et al placed a synthetic dural substitute medial to the fat graft as an inlay. 28 Fibrin glue (e.g., Tisseel or autologous glues) or BioGlue was also used to tack the fat graft in place. 28 29 40

A dural sling describes a material (autologous or synthetic) that is sutured into the dural edges, forming a “sling” or “hammock” that can suspend the resection cavity contents (e.g., fat). The intent of such a technique is to convert a large dural defect with “high-flow” CSF state into a “low-flow” state, as well as reduce compression of risk structures through suspension. Dural slings were performed in two studies, 22 45 and both autologous (fascia) and synthetic materials options were described. Liu et al 22 compared CSF leak rates between synthetic and fascial slings and found a statistically insignificant greater leak rate (20%) with synthetic grafts, notably with a small cohort of eight and five patients for the autologous and synthetic groups, respectively. In both studies, fat was packed into gaps remaining between the graft and dura (fat plugging). Fibrin sealant (e.g., Tisseel) or an onlay of absorbable hemostat (e.g., Surgicel) was also layered onto the sling to improve the seal.

Several studies reported an onlay technique to seal the dura, employing autologous grafts, xenografts, and synthetic dural substitutes. 17 21 22 25 42 46 47 48 49 Autologous onlays consisted of fascia grafts, harvested either from the abdomen or thigh. 17 22 25 42 47 50 Variations in the autologous onlay technique centered around the size of the graft used, ranging from slightly larger than the dural defect to a “peri cranial graft basket.” 46 Advantages of autologous grafts include their cost profile, universal availability, and high compatibility with the local tissue environment, while drawbacks include donor site–related complications and increased operative times. 42 51 A porcine-based small intestinal submucosal xenograft onlay was described in two studies 25 28 which was described as an improvement, as it obviates the need for an autologous fascial harvest (performed from the presigmoid soft tissue at the authors' institutions) which would improve the musculoperiosteal closure. 25 52 Totten et al compared the porcine xenograft closure to a cohort of fascia-based duraplasties and found no difference in CSF leaks. 25 Synthetic onlays consisted of cellulose matrix substitutes (e.g., Duragen, Duramatrix, Duraform, Surgicel) or compressed gelatin sponge (e.g., Gelfoam). Synthetic onlays avoid graft site complications associated with autografts and are resorbable, quickly providing an effective seal. 25 47 48 49 One ineffective synthetic onlay was that described by Arriaga and Chen, 21 consisting of a hardened patty of hydroxyapatite cement (HAC), which was associated with higher CSF leak rates in the first seven patients (28.5%) and was hence discontinued for their remaining cohort. Fibrin glue (e.g., Tisseel) was additionally used to augment the onlay seal in at least three studies. 46 47 50

### Middle Ear Cleft (Eustachian Tube, Middle Ear, and the Aditus ad Antrum)

Despite inconsistent reporting, the treatment of the ET, ME, and aditus was a significant source of variance across studies. First, studies differed by their surgical approach to the ME, which was either by the facial recess approach or via the mastoid. The former involves drilling between the facial nerve and chorda tympani, improving visualization of the ME and ET to enable more precise packing. 26 The latter reaches the ME through the mastoid via holes made during the drilling of the antrum. However, there is equipoise 19 26 whether the facial recess approach indeed improves the seal by facilitating better packing of the ET/ME or if the approach causes a greater flow of CSF into the ME via an unnecessary anatomical corridor, promoting rhinorrhea. 26 35 Overall, it is suggested that the difference in approach is likely only relevant to CSF leak rates in the immediate postoperative period, as the ME is sealed over time from fat grafts in the resection cavity. 53 Christopher et al performed a matched cohort analysis and found no difference in CSF leak rates with the two ME approaches. 26

The majority of studies 15 17 19 20 23 26 27 28 32 33 34 36 38 39 40 41 42 54 55 56 reporting on their treatment of the incus opted to disarticulate and remove it, while at least two studies specified that the incus was left in situ. 19 35 Opponents of removing the incus argue that the technique enlarges the conduit for CSF entry into the ME, while those in favor suggest it allows for more effective packing of the ME and ET. 35 No study compared incus removal in isolation; yet Jacob et al compared the treatment of the ET in two cohorts, one of which did not undergo incus disarticulation—they concluded no difference in CSF outcomes. 19

Twenty-one out of 25 studies reported that the ET could be occluded (or packed), or not treated at all. 15 16 17 18 19 20 23 25 26 27 28 31 32 34 35 36 38 41 48 54 55 56 57 Two studies analyzed the impact of ET closure on CSF rates, both concluding ET packing did not influence CSF leak rates. 19 23 Considering the materials used to pack the ET, the many studies reported the use of muscle, typically harvested from the temporalis or from the sternocleidomastoid. 15 16 17 18 19 20 23 25 26 27 31 32 36 38 55 57 Muscle was combined with fascia, 19 20 23 26 57 oxidized cellulose (Surgicel), 17 19 25 26 27 36 38 or tissue glue. 16 17 55 Other alternatives to muscle grafts for ET packing were polytetrafluoroethylene and carbon filaments (Proplast), 19 high-molecular-weight polyethylene (Plastipore), 19 Surgicel, 19 41 dry periosteum, 17 32 34 54 56 or the incus. 28 SRS packs the ET using bone wax under direct vision. Jacob et al compared various ET packing materials and identified a significant risk of extrusion with Proplast (3%). 19 Studies not directly packing the ET (and ME) cited the benefit as a reduced risk of tympanic membrane injury. 35 Some studies would pack the ME or ET if there was a high degree of temporal bone pneumatization or if an intraoperative Valsalva maneuver caused CSF to collect in the mastoid antrum. 18 31 32 58

Finally, many studies specifying their treatment of the ME opted for packing. 16 18 19 20 23 24 25 26 28 30 31 32 33 34 35 37 38 39 40 41 49 54 55 57 58 59 Materials used for packing included autologous grafts (muscle, 16 18 19 20 21 23 24 25 26 28 30 31 35 39 41 45 48 49 57 fat, 40 dry periosteum 32 33 36 42 47 54 ) and synthetic substitutes (e.g., HAC, 20 30 45 bone wax with or without bone dust, 37 39 55 58 59 and oxidized cellulose 21 25 48 ). Tissue glue was used as an adjunct in at least two case-series. 16 55 Importantly, Sen et al reviewed the use of BioGlue for ME and ET closure, and identified unacceptably high CSF leak rates (62.5%), as such the product was discontinued for this case. 16 Some institutions treated the aditus ad antrum, using combinations of muscle, fat, periosteum, fascia, wax, bone cement. 22 29 39 40 42 54 57

### Air Cells

Air cell treatment included the use of autologous grafts (fat or muscle) and/or synthetic substitutes (e.g., bone wax and HAC). 17 22 23 24 28 29 30 32 33 34 35 36 39 42 47 49 50 54 55 59 60 As with other autologous materials, fat and muscle are widely available yet have associated donor-site complications and risk dislodgement when used in isolation for air cell packing. As such, fat or muscle was premixed with fibrin glues (e.g., Tisseel) prior to application when used in isolation. 17 35 36 Of the synthetic alternatives, bone wax was the most common material reported in the treatment of air cells, and was either used in isolation or in conjunction with bone dust, 39 fat, 28 40 or bone pate and tissue glue. 17 22 23 28 29 32 33 34 39 40 47 49 50 54 55 59 Bone wax is widely used in neurosurgery, and is beneficial due to its effective mechanical tamponade which is cost-effective and provides an immediate seal. 61 An alternative to bone wax is HAC, which may be directly injected into air cells to augment closure. While HAC is more costly compared with bone wax, its liquid consistency enables an effective seal of air cells without a surgeon manually pressing wax into the air cell spaces. 22 24 30 42 Overall, no study compared air cell treatment materials and techniques were frequently combined on an ad hoc basis.

### Translabyrinthine Bony Defect

Following the TL approach to the CPA, petrous bone drilling and mastoidectomy form a dead space that must be reconstructed to prevent CSF leaks and aesthetic defects. Options included autologous grafts (e.g., bone dust or fat) and synthetic alternatives (rigid plate structures, bone cement).

Concerning autologous reconstruction materials, although bone dust would have the biocompatibility and ability to promote bone growth, it increases operative time, donor-site complications, and is limited by quantity. 62 63 As such, the most commonly used autologous graft used to fill the craniectomy dead space is abdominal fat, which was reported in a majority of studies. 15 16 17 18 20 22 23 24 25 26 27 28 29 31 32 33 34 35 36 37 38 39 41 42 45 47 48 49 50 54 55 56 57 58 59 64 Fat graft advantages include long-term durability, revascularization, plentiful abundance, excellent compatibility, and the lack of artifact on postoperative surveillance imaging. 65 However, fat may atrophy over time, resulting in cosmetic defects, and risks dislodgement if insufficient pressure is sustained on its architecture. To keep the graft in place, materials such as biological glue 55 and human fibrin sealant 29 are used to secure it in place. Alternatively (or additionally), fat-filled resection cavities may be combined with rigid plate solutions or HAC.

Rigid plate solutions attempt to solve the fat-associated cosmetic drawbacks by replicating the cranial contours. Additionally, rigid plates act as a buttress by sustaining medial pressure on the fat architecture, improving the watertight seal. Such solutions either consist of a replaced autologous vascularized bone flap or a synthetic plate. The former was reported in one study, 29 while the latter was reported in at least 12 studies. 15 22 25 26 27 41 42 45 47 48 49 50 Regarding synthetic cranioplasties, options include permanent titanium plates and resorbable polyester plates. Titanium is a malleable yet tensile material, allowing the implant to be molded to the cranial contours. However, titanium has the drawback of reduced biocompatibility, for which the coating with inert porous polyethylene (Medpor) may be useful, as it promotes tissue growth into the implant. 22 45 Alternatives to titanium include resorbable plates composed of materials such as poly-(d,l)-lactide 15 48 49 (e.g., rapid resorbable fixation, 48 Resorb X 48 ). Such systems reabsorb within weeks, providing a rigid buttress during the formative stages of wound healing without persisting beyond a few months. Finally, autologous bone cranioplasties, as described by Yuen et al, 29 maintain cosmesis and fat compression while avoiding costly synthetic implants. The authors note limitations of bone flaps include the risk of resorption and donor-site morbidity and specify that the technique is unsuitable for small contracted mastoid cavities or those with anatomical variants such as a laterally placed sigmoid sinus. 29

An alternative or adjunct to fat packing of the TL cavity is reconstruction with HAC. 20 21 24 36 38 46 47 50 Proponents of hydroxyapatite praise its tensile strength, enabled through osteoblastic stimulation, as well as improved cosmesis and reduced tissue traction. 24 Additionally, if HAC is used in isolation, the material obviates the need for an autologous tissue harvest, thus reducing operative times and donor-site morbidities. Opponents of the HAC cranioplasty raise concerns, including the risk of delayed extrusion, 66 and high cost ($4,000–$7,000 per operation 30 ). HAC can replace fat in the temporal resection cavity or be used to augment fat and titanium cranioplasties. 47 50 The benefit of the combined use of fat, HAC, and a titanium mesh is to gain from the HAC's cosmetic properties, while enabling expeditious removal of the mesh (and HAC) should re-entry to the CPA be required. There were five studies 20 21 24 36 38 (from three centers) comparing HAC cranioplasties to same-center historical cohorts of fat cranioplasties. The largest study was a mixed prospective/retrospective study evaluating 369 HAC cranioplasties, and found the technique reduced CSF leaks compared with fat graft solutions ( *p* < 0.001). Sioshansi et al and Arriaga et al found comparable rates of CSF leak between fat and HAC cranioplasties, yet reported improved postoperative headaches with HAC. 20 38 Similarly, Hussain and Ahsan 46 modified the HAC closure technique by interposing a thin layer of porcine-based sterile sponge (Gelfoam) between the cement and soft tissue which also reduced headaches. Overall, despite early warnings of HAC use in skull base reconstruction, 66 no study demonstrated the inferiority of HAC compared with AFG for the TL approach.

### Extracranial Soft Tissue Closure

Techniques varied by the number of layers used to close the extracranial soft tissue. Fat-filled resection cavities, without rigid buttress solutions, rely on the medial pressure achieved through the tight closure of the periosteum, fascia, and muscle to maintain a tight architecture. The periosteal layer was either closed individually 15 20 27 40 42 47 50 or together with muscle/fascia. 16 18 24 25 28 31 32 33 37 55 Fishman et al specified the use of a “Palva Flap,” which is a broad-based periosteal flap on an anterior pedicle. 40 Yuen et al 29 used a vascularized mastoid bone flap to medially compress the fat-filled resection cavity, whereby the periosteum was never elevated from the mastoid cortex. Although rarely specified, sutures were closed in an interrupted or running fashion and the materials used included 2–0 Vicryl. 27 The skin was closed with staples, 31 46 3–0 Prolene, 27 Nylon, 48 49 2–0 Vicryl, 17 or Silk 32 using either interrupted or running interlocking techniques. Moderie et al 17 described the injection of human fibrin sealant in between the subcutaneous tissue and the temporal muscle, as well as applying a supra-auricular absorbable suture through all closure layers to improve the watertight seal. No study performed a comparative analysis of soft tissue closure techniques.

### Dressings

Some groups regard postoperative dressings as an important consideration in the prevention of postoperative leaks. Mastoid pressure dressings may apply medial pressure to the dead space reconstructive materials, potentially reducing the incidence of postoperative leaks. 17 24 27 29 33 35 42 64 When specified, the duration of application of such dressings ranged from 48 hours 27 29 to 192 hours. 33

### CSF Diversion

Whether lumbar drains were used during TL resections was only specified in 12 studies. Ten studies specified that CSF diversion was not performed. 16 22 23 26 27 31 33 35 39 64 Ölander et al placed a preoperative LD for grade IV tumors to manage intracranial pressure pre- and postoperatively. 56 Crowson et al placed elective lumbar drains in 110/121 of their TL cohort and reported no difference in CSF leak rates. 12 The mechanism of CSF leaks suggests that prophylactic lumbar drains may act beneficially by reducing the pressure gradient across the skull. Alternative purposes of intraoperative lumbar drains include improving the exposure by aiding drainage, which relaxes the intracranial contents. However, lumbar drains have associated complications related to the rate of CSF drainage (e.g., low pressure headaches), catheter failure, and infections. 12 Overall, no included study reported that lumbar drains reduced the incidence of CSF leaks.

### CSF Leaks and Their Management

CSF leaks were defined as rhinorrhea, otorrhea, or incisional leaks. The median CSF leak rate across studies was 6% (IQR: 1–10%). Due to overlap in patient cohorts and variable analysis criteria, such as the exclusion of deceased patients by Volsky et al, 36 a pooled synthesis of CSF leaks was omitted. The median rhinorrhea, otorrhea, and incisional leak rates were 2% (IQR: 0–4%), 0% (IQR: 0–3%), and 0% (IQR: 0–0%), respectively. There were three cohorts of patients (19 patients in total) that were significant outliers regarding higher CSF leak rates. Sen et al's study assessed the use of BioGlue for ME cleft stage treatment and found a leak rate of 62.5% in the first seven patients—leading to a discontinuation of BioGlue for this use case. 16 Moreover, Arriaga et al's 2002 analysis of a hardened HAC patty onlay resulted in high leak rates (29%) and thus was also discontinued for the remaining cohort. Finally, Liu et al explored the impact of a synthetic fascial sling for the dural treatment stage and found one leak in five patients. Excluding these three cohorts (19 patients) reduces the maximum overall CSF leak rate experienced in the remaining 8,494 patients to 16%. 22 While only specified in four studies, CSF leaks were confirmed based on clinical symptoms and signs (rhinorrhea, otorrhea, incisional leak) or through biochemical tests (glucose 23 or β2-transferrin 57 ). The standard operating procedure employed when a leak was suspected or confirmed was inconsistently reported, yet consisted of a graded escalation protocol, starting with conservative measures (absolute bed rest, avoiding coughing, compressive dressings) followed by various intermediate strategies (lumbar punctures, 55 wound re-suturing, 26 acetazolamide 23 ) and finally surgical repair. Twenty-nine of 43 studies performed at least one surgical repair. 15 16 18 20 22 23 24 25 27 30 32 33 34 36 37 38 39 40 41 42 45 48 49 54 55 57 58 59 64

## Discussion

### Principal Findings

We conducted a systematic review of 43 papers examining the various operative protocols used during TL VS surgery. The motivation for the work was to progress the understanding of the ideal protocol by which to prevent postoperative CSF leaks following TL VS surgery. CSF leaks have several associated morbidities, including low-pressure headaches, pneumocephalus, and life-threatening meningitis. Additionally, CSF leaks may necessitate repeat operations, leading to increased healthcare costs and lengthened hospital stays. Chern et al estimated the median cost of repairing a CSF leak as $50,401. 7 While multiple risk factors are associated with CSF leaks, the intraoperative repair protocol is of particular significance. This review classifies the repair methods into seven stages, including the dura, ME cleft, air cells, TL skull resection cavity, extracranial soft tissue, dressing application, and CSF diversion. The main findings are detailed below.

Across the seven stages in the operative protocol for preventing CSF leaks, authors described various techniques using autografts, xenografts, and synthetic materials. The dural stage was specified in 32 studies, of which the most common technique was fat plugging. Alternatives included the use of autografts (e.g., fascia, muscle, pericranium), xenografts (e.g., small intestinal mucosa), or synthetic materials (e.g., oxidized cellulose, collagen, gelatin) for onlays, inlays, and sutured slings. Overall, 19 different combinations of materials and techniques were reported. The ME cleft treatment stage considers the combined approach to the incus, ME, ET, and aditus, which were repaired using autologous grafts (e.g., muscle, fascia, fat, bone dust) and synthetic materials (e.g., cellulose, bone wax, bone cement, glues). One important non-compatible material for the ME stage was the use of BioGlue, which was identified by Sen et al as having unacceptably high leak rates (62.5%). 16 In total, the ME approach was described in 32 studies and resulted in 34 combinations of techniques and materials. The air cell stage was described in 21 studies and most commonly involved the obliteration of potential CSF tracts with bone wax (described in 12/21 studies) with or without mucosal stripping and endoscopic visualization. The alternative biomaterials used in the air cell stage included muscle, bone cement, fat, bone dust, periosteum, and glue. The TL resection cavity involved the packing of the defect, typically with strips of abdominal fat without a bony skull reconstruction (craniectomy). Key alternatives involved the applications of synthetic skull substitutes composed of either titanium or resorbable plates. There were 12 unique approaches to the TL resection cavity. Dressings varied based on whether they applied medial pressure to the bony repair and the duration of application. The extracranial soft tissue varied by the number of layers to close the skin and the suture materials used. CSF diversion strategies were specified in 12 studies and usually not performed.

A substantial degree of variation was observed both within and between institutions. Concerning the latter, every group employed a unique combination of materials and techniques across closure stages. Stages that were relatively homogenous between studies included the extracranial soft tissue stage and the CSF diversion approach. On the contrary, the dural stage and ME stage were sources of greater heterogeneity, as there were 19 and 34 combinations of techniques and materials described in 32 studies each. Stages with a moderate degree of heterogeneity were the TL resection cavity stage and air cell stage, as most centers obliterated the spaces using bone wax and fat, respectively, resulting in 12 and 10 combinations from 38 and 23 studies, respectively. Additionally, there was intra-institutional heterogeneity of repair techniques, exemplified by the several studies describing interchangeable techniques for dural closure stages, using either autologous or synthetic materials, without specifying any considerations that would favor one material over another. Indeed, this highlights the uncertainty associated with the operative repair protocols.

As outlined in part 1 of this series, the lack of consensus regarding which technique best prevents CSF leaks is likely due to the lack of high-level evidence. Indeed, the number of patient, lesion, and operative factors influencing the incidence of CSF leaks makes it difficult to draw conclusions from a single institution's retrospective analysis. This is further complicated by the inconsistent reporting of key data points and the likely complexity of interaction between factors. To address this issue, we suggest a novel study design in the form of a prospective, multicentered service evaluation capturing a comprehensive dataset of potential CSF leak risk factors across multiple domains, including operative protocols. More specifically, the operative data capture should address all the repair stages outlined in the present study. Such study designs have previously enabled the effective comparison of endonasal repair protocols following endoscopic endonasal surgery and identified strategies associated with a greater propensity of leak-free surgery. 67

### Findings in Context

To our knowledge, the only other systematic review of prophylactic skull base repair strategies is the meta-analysis by Selesnick et al, performed in 2004. 68 In their review, they identified similarly popular techniques for certain repair stages, namely bone wax obliteration of air cells, and free fat packing of the TL defect. Additionally, the obliteration of the eustachian was identified as a popular technique, yet without a demonstrated impact on leak rates. Overall, they also could not comment on the superiority of different operative protocols. Our study expands on that by Selesnick et al, as it provides a comprehensive categorization of repair techniques, and identifies the multistaged heterogeneity that separates the protocols employed at different institutions. As such, we caution against the pooled comparison of different protocols, as the current evidence, which is both heterogenous techniques and reporting, is difficult to synthesize. Additionally, we expand on previous work by Layard Horsfall et al in which a codified, operative workflow was established for not only the closure phase of the TL approach but all 59 steps of the operation. 69 More specifically, we highlight the heterogeneity in international practice within the phases delineated in their workflow and expand on the biomaterials used in the repair of the lateral skull base.

### Strengths and Limitations

In this systematic review, we utilized a structured approach to generate a broad and comprehensive overview of surgical practice pertinent to TL VS surgery. Through international inclusion criteria, limited to the most recent 23 years of surgical practice, we achieved a pragmatic balance within a comprehensive scope that remained relevant to modern-day surgical practice.

To highlight salient variations, we developed a taxonomy to classify the diverse repair protocols into seven distinct stages. Nonetheless, this classification system may not adequately convey the potential for individual repair techniques to have a multi-staged impact. For instance, the choice of materials, such as free fat grafts, utilized for filling the bony TL defect can influence the seal achieved at other stages, such as the dura or air cells.

It is important to acknowledge the methodological limitations of the included studies, as most were characterized by observational and retrospective designs, which inherently pose risks such as selection bias, information bias, measurement errors, and confounding variables. Additionally, it is worth noting that studies reporting surgical outcomes are frequently susceptible to publication bias.

Finally, the inconsistent reporting of critical outcome measures across studies, including a range of CSF leak predictive factors, precluded a comparative meta-analysis of the repair protocols.

## Conclusion

Internationally, the intraoperative approaches aimed at averting CSF leaks following TL VS surgery exhibit significant heterogeneity across institutions. In this systematic review, we propose a taxonomy of seven stages, categorizing operative techniques and materials intended to prevent CSF leaks. However, we highlight the complexity of comparing these techniques due to the substantial number of predictive factors for CSF leaks, coupled with inconsistent reporting of these factors. To advance our understanding and management of these leaks, future service evaluations must adopt a prospective approach, capturing a holistic selection of potential predictive factors for CSF leaks, including all stages of the lateral skull base repair protocols.
