# Supplementary material for: Skull Base Repair following Resection of Vestibular Schwannoma: A Systematic Review (Part 2: The Translabyrinthine Approach)
Source: J Neurol Surg B Skull Base. 2024 Jan 22;85(Suppl 2):e131–44. doi: 10.1055/a-2222-0016 (PMC11495917; doi:10.1055/a-2222-0016)
Supplement: Supplementary file 1 — Supplementary Material [file 10-1055-a-2222-0016-s23sep0148.pdf]

Supplementary Table S1 Search strategy

Term	Keywords
1	(Retrosigmoid or translabyrinthine or "vestibular schwannoma" or "lateral skull base" OR "acoustic neuroma").mp. [mp = ti, ab, hw, tn, ot, dm, mf, dv, kf, fx, dq, bt, nm, ox, px, rx, ui, sy, ux, mx]
2	("Cerebrospinal fluid" or CSF or leak or pyorrhoea or rhinorrhoea or "wound leak" or otorrhoea).mp. [mp = ti, ab, hw, tn, ot, dm, mf, dv, kf, fx, dq, bt, nm, ox, px, rx, an, ui, sy, ux, mx]
3	1 AND 2

Supplementary Table S2 Risk of bias and quality assessment

Bias domains and scoring:

A. Description of patient: detail provided of patient characteristics.
   i. High risk of bias (scored 1): No description of patient demographics and CSF leak risk factors (i.e., patients underwent VS resection).
   ii. Low risk of bias (scored 0): Clear description of patient demographics, including potential risk factors (i.e., BMI, tumor size, etc.).

B. Description of pathologies: detail provided of lesions.
   i. High risk of bias (scored 1): No reporting of cohort pathology frequency (i.e., VS, meningioma, and other).
   ii. Low risk of bias (scored 0): Clear description of pathology frequency (i.e., VS = 100, meningioma = 5).

C. Description of repair technique I: identifiability of each treatment group.
   i. High risk of bias (scored 1): Heterogenous repair strategies without description of frequency used (i.e., repair technique A or B or C was used based on surgeon preference).
   ii. Low risk of bias (scored 0): Description in which percentage/proportion of cases a repair technique was used (e.g., repair A was used for tumors >2.5 cm (n = 6) or repair B was used in all cases).

D. Description of repair technique II: clarity of description of technique and materials.
   i. High risk of bias (scored 1): Minimal detail of repair technique described (e.g., dura was closed using fascia).
   ii. Low risk of bias (scored 0): Clear description of all stages of skull base repair (dura, IAC, air cells, skull, and soft tissue).

E. Clarity of outcomes: Clarity of definition of CSF leaks.
   i. High risk of bias (scored 1): No description of CSF leak term diagnosis (e.g., CSF leaks were identified).
   ii. Low risk of bias (scored 0): Site of CSF leak provided (e.g., rhinorrhea, incisional leak, otorrhea) or reported separately from pseudomeningoceles.

Scoring per paper:

Author	Year	PMID	A	B	C	D	E	Total
Martinez Perez	2021	**34642820**	1	1	1	0	0	3
Freeman et al	2022	35671137	0	0	1	0	0	1
Plainfossé et al	2021	33942123	1	0	1	0	0	2
Cooper et al	2021	33997716	1	0	1	0	0	2
Totten et al	2021	34745839	0	0	0	0	0	0
Christopher et al	2021	34121082	1	0	0	1	1	3
Selleck et al	2021	33443978	0	0	0	1	0	1
Luryi et al	2019	30843619	1	0	0	0	0	1
Jacob et al	2015	26208128	0	0	1	1	1	3
Boghani et al	2014	23371869	1	0	0	1	0	2
De Campos Netto et al	2012	24083124	1	0	1	0	1	3
Ammar et al	2012	22270232	1	0	0	1	0	2
Angeli et al	2011	21131891	1	0	0	0	0	1

(*Continued*)

 © 2024. The Author(s).

(*Continued*)

Author	Year	PMID	A	B	C	D	E	Total
Hillman et al	2011	21817943	1	0	0	0	1	2
Goddard et al	2010	20084041	1	0	0	0	0	1
Bambakidis et al	2010	21318032	1	0	0	1	0	2
Yuen et al	2009	19881899	1	0	0	0	0	1
Arriaga et al	2007	17414174	1	0	0	1	0	2
Volsky et al	2017	28059442	1	0	1	1	0	3
Russel et al	2017	27851657	1	0	0	1	1	3
Sioshansi et al	2021	34411065	0	0	0	1	1	2
Manjila et al	2013	23340781	1	0	0	0	0	1
Zhang et al	*2012*	*23147594*	*1*	*0*	*1*	*1*	*1*	*4*
Liu et al	*2012*	*22937851*	*1*	*0*	*0*	*0*	*0*	*1*
Charpiot et al	*2009*	*20443757*	*1*	*0*	*0*	*1*	*1*	*3*
Roche et al	*2008*	*18810201*	*1*	*0*	*0*	*1*	*0*	*2*
Olander et al	*2017*	*29361875*	*1*	*0*	*0*	*1*	*0*	*2*
Moderie et al	*2017*	*28816691*	*1*	*0*	*0*	*0*	*0*	*1*
Brennan et al	*2001*	*11213957*	*1*	*0*	*0*	*1*	*0*	*2*
Arriaga et al	*2002*	*12075225*	*1*	*0*	*0*	*1*	*1*	*3*
Leonetti et al	*2001*	*11337653*	*1*	*1*	*1*	*1*	*1*	*5*
Wiet et al	*2009*	*10892703*	*1*	*0*	*0*	*0*	*0*	*1*
Becker et al	*2003*	*12544038*	*1*	*0*	*0*	*1*	*0*	*2*
Sanna et al	*2003*	*14738606*	*1*	*0*	*0*	*0*	*1*	*2*
Khrais et al	*2009*	*15179205*	*1*	*0*	*0*	*0*	*0*	*1*
Kalamarides et al	*2004*	*15354009*	*1*	*0*	*1*	*0*	*0*	*2*
Fishman et al	*2009*	*15091225*	*1*	*0*	*0*	*0*	*0*	*2*
Cueva et al	*2005*	*16272937*	*1*	*0*	*0*	*1*	*1*	*3*
Sen et al	*2006*	*16371855*	*1*	*0*	*0*	*1*	*0*	*2*
Akhtar et al	*2005*	*15794544*	*1*	*0*	*0*	*1*	*0*	*2*
Fayad et al	*2007*	*17414045*	*1*	*0*	*0*	*1*	*0*	*2*
Jacob et al	*2007*	*17704723*	*1*	*1*	*0*	*1*	*1*	*4*
Crowson et al	*2015*	*26239000*	*1*	*0*	*0*	*1*	*1*	*3*

 © 2024. The Author(s).

Supplementary Table S3 Translabyrinthine repair techniques classified according to stages of repair, ordered by CSF leak %

Authors	Patients	CSF leaks (*n*)	CSF leak %	Dural	Middle ear cleft	Air cells	TL resection cavity	Soft tissue	CSF diversion
Plainfosse et al 2021	8	0	0.0%	Temporalis fascial graft (technique NS)	NO facial recess approach + ET NOT packed + AAA packed (fascia)	Sealed (BW)	Packed with abdominal flank fat.	Operative wound was closed in layers	Not performed
Selleck et al 2021	38	0	0.0%	Fat packing	Facial recess approach + incus removed + ET packed (Surgicel and muscle)	NS	Packed with strips of fat	WT periosteal flap (running 2–0 Vicryl) + tight s/c layer (interrupted 2–0 Vicryl + skin (running interlock 3-0 Prolene)	Lumbar drains were not universally utilized
Goddard et al 2010	61	0	0.0%	Fat packing	Aditus approach + incus NOT removed + ET NOT packed + ME packed (muscle and fascia)	Sealed (muscle)	Packed with fat	Periosteal layer + s/c layer + skin (nonabsorbable suture)	Not performed
Bambakidis et al 2010	15	0	0.0%	Fascial onlay + fibrin glue	NS	Sealed (BW)	Packed with abdominal fat + Titanium mesh plate + HAC	WT periosteum + “usual layered closure of soft tissue” followed by the usual layered closure of soft tissue	NS
Heng-wai et al 2009	27	0	0.0%	Fat packing + fibrin glue	Aditus ad antrum packed with temporalis fascia and wax	Sealed (BW)	Packed with fat + fibrin glue + vascularized bone flap	Vascularized mastoid bone flap with thick layer of musculoperiosteum is raised and replaced through suture suspension + “standard wound closure”	NS
Heng-wai et al. 2009	10	0	0.0%	Fat packing + fibrin glue	Aditus ad antrum packed with temporalis fascia and wax	Sealed (BW)	NS	Deformable reconstruction	NS
Manjila et al 2013	42	0	0.0%	Fascial onlay + fibrin sealant + Gelfoam onlay + fibrin glue (Tisseel)	NS	Sealed. (BW)	Packed with abdominal fat+ titanium mesh plate + bone cement	WT periosteal closure + “standard closure of soft tissue”	NS
Liu et al 2012	8	0	0.0%	Fascial sling + fascial IAC onlay (abdomen/thigh) + Surgicel suture line onlay	AAA packed (muscle + bone cement—HydroSet)	Sealed (BW or HAC)	Fat graft + fibrin glue + Surgicel + fat + titanium mesh plate embedded in porous polyethylene (Medpor)	Layered closure	Not performed
Arriaga et al 2002	47	0	0.0%	Fat packing	NS	NS	HAC	NS	NS
Akhtar et al 2005	20	0	0.0%	Pericranial graft basket onlay + tissue glue	NS	NS	HAC + Gelfoam	Closed in two layers (absorbable sutures and staples)	NS
Sanna et al 2003	596	5	0.8%	Fat packing	Incus removed + ME packed (dry periosteum) + IF highly pneumatized bone ET packed with muscle pieces	Mucosa removed from air cells + sealed (BW)	Packed with abdominal fat (strips)	Fasciomusculoperiosteal flap + skin fixed to underlying layer with 2-3 absorbable suture + skin closed with silk sutures	NS
Mehdi et al. 2012	1865	16	0.9%	Fat packing + fascial sling sutured into dura	Aditus approach + incus removed + ET packed (dry periosteum) ME packed (dry periosteum)	Sealed (BW)	Packed with long thin fat strips	NS	NS
Arriaga et al 2017	90	1	1.1%	Fat packing	Incus removed + ET packed (muscle + fibrillar surgical) + ME packed (muscle + fibrillar surgical)	NS	Packed with fat strips + HAC (bone source)	NS	NS

(*Continued*)

 © 2024. The Author(s).

Supplementary Table S3 (*Continued*)

Authors	Patients	CSF leaks (*n*)	CSF leak %	Dural	Middle ear cleft	Air cells	TL resection cavity	Soft tissue	CSF diversion
Khrais et al 2009	710	10	1.4%	Fat packing	Aditus approach + incus removed + ME packed (dry periosteum)	Mucosa removed + sealed (BW). Endoscope used to check air cells, especially infralabyrinthine air cells	Packed with abdominal fat (strips)	Musculofascioperiosteal flap + skin and sc tissue flap	No intraoperative CSF diversion via LD
Cueva et al 2005	126	2	1.6%	Dural approximation + fat packing	NS	Sealed (BW)	Packed with fat strips	The muscle layer is closed with interrupted sutures of 2–0 woven polyglactic acid suture spaced approximately 1 cm or less apart. The deep cutaneous layer is closed with inverted interrupted sutures of the same material similarly spaced	Lumbar drainage of CSF was not used perioperatively
Angeli et al. 2011	110	2	1.8%	NOT CLOSED	Incus removed + ET packed (dry periosteum) + ME packed (dry periosteum)	Sealed (BW)	Packed with long strips of abdominal fat	NS	NS
Hunter et al 2015	53	1	1.9%	Synthetic dural onlay (Duraform)	ET packed (muscle and Surgicel) + ME packed with muscle and Surgicel	NS	Packed with strips of abdominal fat graft + resorbable mesh plate (DePuys/ResorbX)	"s/c tissue and skin closed in 3 layers." Skin closed with running locking nylon or staples	NS
Volsky et al 2017	369	7	1.9%	Fat packing	NO facial recess approach + incus removed (sometimes) + ET packed sometimes (muscle and Surgicel) + antrum packed (muscle)	Sealed (muscle)	Packed with fat strips + HAC (HydroSet)	NS	NS
Moderie et al 2017	94	2	2.1%	Temporalis fascia onlay	Facial recess approach + incus removed + ET Packed (musculoperiosteum, BW, fibrin sealant, oxidized cellulose-Surgicel)	Sealed (fat + human fibrin sealant)	Packed with fat + human fibrin sealant	Incision closed (watertight) + human fibrin sealant injected between the s/c tissue and temporal muscle + A supra-auricular, transcutaneous, and transmuscular 2.0 Vicryl suture is placed to ensure a tight closure between the skin and the temporal muscle	NS
Wiet et al 2009	44	1	2.3%	fat packing	Incus removed + facial recess NOT opened + ME packed (muscle) + AAA packed (BW covered in bone dust)	Sealed (BW + bone dust)	Packed with abdominal fat	Three layered closure of skin and s/c tissue	Not performed
Sioshansi et al 2021	33	1	3.0%	Approximation with temporalis fascia, Gelfoam, or dural graft matrix	Incus removed + ET packed (fascia + muscle) + ME packed (bone cement—HydroSet)	NS	HAC	Periosteal layer approximated (absorbable suture) + WT s/c tissue + skin closure	NS
Fayad et al 2007	389	13	3.3%	Dural approximation + fat packing	Incus removed + ET packed (Surgicel) + ME packed (muscle)	NS	Packed with fat+ titanium mesh plate	NS	NS

 © 2024. The Author(s).

Supplementary Table S3 (*Continued*)

Authors	Patients	CSF leaks (*n*)	CSF leak %	Dural	Middle ear cleft	Air cells	TL resection cavity	Soft tissue	CSF diversion
Leonetti et al 2001	209	8	3.8%	Fat packing	Valsalva -> If CSF collected in the antrums, but the hearing was lost -> ET and ME packed via facial recess approach (temporalis muscle) + AAA packed (BW+ bone pate)	NS	Packed with fat	Musculoperiosteal flap+ scalp repaired (running locking suture OR staples)	Not performed
Luryi et al 2019	52	2	3.9%	NOT CLOSED	ME packed (bone cement)	Sealed (HAC)	Packed with HAC	s/c layer reapproximated + skin reapproximated	NS
Cooper et al 2021	23	1	4.4%	Primary approximation + fascial onlay OR fat packing	Incus removed (sometimes) + ME packed (periosteum, fascia, tendinous tissue) + aditus packed (periosteum, fascia, tendinous tissue) + fascial graft over aditus	Sealed (periosteum + fascia + HAC)	Packed with strips of fat +- titanium mesh plate	Watertight closure periosteal/fascia layer + tight closure s/c layer + skin closure	NS
Totten et al 2021	21	1	4.8%	Synthetic dura onlay (Duraform) + 2 layers of small intestine graft onlay	ET packed (muscle and Surgicel) + ME packed (muscle and Surgicel) attic repaired with bone pate + fascia	NS	Packed with fat+ resorbable mesh plate	WT closure musculoperiosteal flap + WT closure subcutaneous layer + skin	NS
Fishman et al 2009	101	5	5.0%	Fat packing + primary dural approximation + fibrin glue	Incus removed + ME packed (fat) + aditus packed (fat)	Sealed (BW + fat)	NS	Pedicled periosteal "Palva flap" + "layered closure"	NS
De Campos Netto et al 2012	18	1	5.6%	Fat packing	Facial recess approach + incus removed + ET packed (using incus) + ME packed (muscle)	Sealed (BW + fat)	Packed with fat strips	Musculoperiosteal flap (tight) + s/c (tight) + skin (tight)	NS
Kalamarides et al 2004	139	8	5.8%	NS	ME packed (muscle) + ET packed if highly pneumatized bone (muscle)	NS	Packed with abdominal fat (strips)	Periosteomuscular flap + skin	NS
Jacob et al 2007	51	3	5.9%	NS	Incus NOT removed + ET NOT packed + ME packed (muscle) + aditus covered (fascia)	NS	NS	NS	NS
De Campos Netto et al 2012	16	1	6.3%	Fat packing, synthetic dural underlay + fibrin glue	Facial recess approach + incus removed + ET packed (using incus) + ME packed (muscle)	Sealed (BW + fat)	Packed with fat strip + 2 dural substitute layers + fibrin glue	Musculoperiosteal layer (tight), s/c tissue (tight) + skin (tight)	NS
Charpiot et al 2009	123	8	6.5%	NS	ME packed (BW)	Sealed (BW)	Packed with abdominal fat + fibrinogen glue	Layered closure	NS
Sioshansi et al 2021	30	2	6.7%	Fat packing	ME packed (muscle), fascia creates seal over aditus and epitympanum	NS	Packed with fat	NS	NS
Zhang et al 2012	115	8	7.0%	Primary (incomplete) approximation	AAA packed (BW) + ME packed if highly pneumatized temporal bone (BW)	NS	Packed with abdominal fat (strips)	NS	NS
Pierre-Hugues et al 2008	110	8	7.3%	Fat packing	ME packed (bone dust + fibrin glue)	NS	packed with abdominal fat	Musculofascial flap (tight) + skin	NS

(*Continued*)

 © 2024. The Author(s).

Supplementary Table S3 (*Continued*)

Authors	Patients	CSF leaks (*n*)	CSF leak %	Dural	Middle ear cleft	Air cells	TL resection cavity	Soft tissue	CSF diversion
Boghani et al 2014	13	1	7.7%	Sutured fascial sling (fascia lata)	Aditus ad antrum packed (small muscle graft) + hydroxyapatite cement	NS	Packed with fat + Titanium Mesh Plate coated with Porous Polyethylene (Medpor)	NS	NS
Christopher et al 2021	102	8	7.8%	NS	Facial recess approach + incus removed + ET packed (muscle, BW, fascia, Gelfoam, oxidized cellulose, abdominal fat) + ME packed (muscle)	NS	NS	NS	Not performed
Jacob et al 2007	148	12	8.1%	NS	Incus removed + ET packed (various) + ME packed (muscle)	NS	NS	NS	NS
Martinez-Perez et al 2021	34	3	8.8%	NOT CLOSED	Incus not removed + ME packed (muscle)	Sealed (HAC) and compressed with neurosurgical patty	Packed abdominal fat strips + HAC.	Deep fascial plane + s/c plane (interrupted absorbable sutures) + skin (running suture)	NS
Ölander et al 2017	700	67	9.6%	NS	Incus removed + malleus removed + ET packed (periosteum and fibrin glue)	NS	Packed with abdominal fat	Vascularized muscle flap + skin closed (sutures)	Patients with grade IV tumors were treated with a ventricular drainage before the TLA was performed
Christopher et al 2021	102	10	9.8%	NS	NO facial recess approach + incus removed + ET packed (muscle, BW, fascia, Gelfoam, oxidized cellulose, fat) + ME packed (muscle)	NS	Packed with abdominal fat + titanium mesh plate.	NS	Not performed
Brennan et al 2001	431	43	10.0%	NS	Incus removed and head of malleus removed + ET packed (temporalis muscle and fascia) + ME packed (temporalis muscle and fascia) + AAA packed (fascia lata)	NS	Packed with fat	Vascularized periosteal flap	NS
Freeman et al 2022	205	21	10.2%	Synthetic dura onlay (Duragen)	Me packed (muscle + Surgicel)	Sealed (BW)	Packed with areolar fat + resorbable polymer plate (KLS Martin)	Skin and s/c tissue closed in 3-layered fashion. Running nylon locking sutures for skin	ND
Martinez-Perez et al 2021	35	4	11.4%	NOT CLOSED	Incus NOT removed + ME packed (muscle)	NS	Packed with abdominal fat strips	Watertight myoperiosteal flap + layered closure + skin	NS
Russel et al 2017	275	33	12.0%	NS	Incus removed + ET packed (muscle+ biological glue) + ME packed (bone pate and biological glue and BW)	Sealed (multilayered bone pate, biological, glue, and wax)	Packed with fat + biological glue.	Musculoperiosteal flap + cutaneous incision closed	NS
Crowson et al 2015	121	15	12.4%	NS	NS	NS	NS	NS	LD for 2 days OR no LD

Supplementary Table S3 (*Continued*)

Authors	Patients	CSF leaks (*n*)	CSF leak %	Dural	Middle ear cleft	Air cells	TL resection cavity	Soft tissue	CSF diversion
Hillman et al 2011	71	9	12.7%	Fat packing	incus removed + ET packed (muscle)	NS	Packed with strips of abdominal fat layered + tight periosteal closure	Periosteum (tight) + "2 water tight layers"	NS
Selleck et al 2021	94	12	12.8%	Fat packing	Facial recess approach + incus removed + ET packed (Surgicel and muscle)	NS	Packed with strips of fat + titanium mesh plate	Residual periosteum sutured over the mesh where possible + tight s/c layer (interrupted 2-0 Vicryl + skin (running interlock 3–0 Prolene)	NS
Arriaga et al 2002	54	7	13.0%	Fat packing	NS	NS	NS	NS	NS
Becker et al. 2003	100	13	13.0%	NS	ET packed (bone wax) + ME packed (muscle)	NS	Packed with fat (strips)	NS	NS
Hillman et al 2011	149	20	13.4%	Fat packing	Incus removed + ET packed (muscle)	NS	Packed with fat + resorbable mesh plate (Synthes)	NS	NS
Plainfosse et al 2021	39	6	15.4%	Temporalis fascial graft (technique NS)	Facial recess approach + Incus removed + ET packed (muscle-fascia) + ME packed (muscle fascia)	Sealed (BW)	Packed with abdominal flank fat.	Operative wound closed in layers	Not performed
Totten et al 2021	56	9	16.1%	Synthetic dura onlay (Duraform) + fascial onlay	ET packed (muscle and Surgicel) + ME packed (muscle and Surgicel) attic repair with bone pate + fascia	NS	Packed with fat + resorbable mesh plate	Watertight closure of musculoperiosteal flap and subcutaneous layer is done before	NS
Liu et al 2012	5	1	20.0%	Dural substitute sling (Duragen/Matrix) + Surgicel suture line onlay	AAA packed (muscle + bone cement—HydroSet)	Sealed (BW or HAC)	Fat graft+ fibrin glue + Surgicel + fat + titanium mesh plate embedded in porous polyethylene (Medpor)	Layered closure	Not performed
Arriaga et al 2002	7	2	28.6%	Bone cement (bone source) onlay	NS	NS	HAC	NS	NS
Aloke et al 2006	24	15	62.5%	Fascia, muscle, strips of abdominal fat (technique NS) + BioGlue	ET packed (temporalis muscle and BioGlue) + ME packed (temporalis muscle and BioGlue)	NS	Packed with abdominal fat	Musculoperiosteal flap	Lumbar drain not used perioperatively

Abbreviations: AAA, aditus ad antrum; BW, bone wax; CSF, cerebrospinal fluid; ET, eustachian tube; HAC, hydroxyapatite cement; LD, lumbar drain; ME, middle ear; NS, not specified; s/c, subcutaneous; TL, translabyrinthine; WT, watertight.
